# AdpA_lin_, a Pleiotropic Transcriptional Regulator, Is Involved in the Cascade Regulation of Lincomycin Biosynthesis in *Streptomyces lincolnensis*

**DOI:** 10.3389/fmicb.2019.02428

**Published:** 2019-10-23

**Authors:** Yajing Kang, Yingying Wang, Bingbing Hou, Ruida Wang, Jiang Ye, Xiaoyu Zhu, Haizhen Wu, Huizhan Zhang

**Affiliations:** ^1^State Key Laboratory of Bioreactor Engineering, East China University of Science and Technology, Shanghai, China; ^2^Department of Applied Biology, East China University of Science and Technology, Shanghai, China

**Keywords:** AdpA, lincomycin, *Streptomyces lincolnensis*, transcriptional regulator, cascade regulation

## Abstract

Lincomycin is one of the most important antibiotics in clinical practice. To further understand the regulatory mechanism on lincomycin biosynthesis, we investigated a pleiotropic transcriptional regulator AdpA_lin_ in the lincomycin producer *Streptomyces lincolnensis* NRRL 2936. Deletion of *adpA*_*lin*_ (which generated Δ*adpA*_*lin*_) interrupted lincomycin biosynthesis and impaired the morphological differentiation. We also found that putative AdpA binding sites were unusually scattered in the promoters of all the 8 putative operons in the lincomycin biosynthetic gene cluster (BGC). In Δ*adpA*_*lin*_, transcript levels of structural genes in 8 putative operons were decreased with varying degrees, and electrophoretic mobility shift assays (EMSAs) confirmed that AdpA_lin_ activated the overall putative operons via directly binding to their promoter regions. Thus, we speculated that the entire lincomycin biosynthesis is under the control of AdpA_lin_. Besides, AdpA_lin_ participated in lincomycin biosynthesis by binding to the promoter of *lmbU* which encoded a cluster sited regulator (CSR) LmbU of lincomycin biosynthesis. Results of qRT-PCR and catechol dioxygenase activity assay showed that AdpA_lin_ activated the transcription of *lmbU*. In addition, AdpA_lin_ activated the transcription of the *bldA* by binding to its promoter, suggesting that AdpA_lin_ indirectly participated in lincomycin biosynthesis and morphological differentiation. Uncommon but understandable, AdpA_lin_ auto-activated its own transcription via binding to its own promoter region. In conclusion, we provided a molecular mechanism around the effect of AdpA_lin_ on lincomycin biosynthesis in *S. lincolnensis*, and revealed a cascade regulation of lincomycin biosynthesis by AdpA_lin_, LmbU, and BldA.

## Introduction

Lincomycin is a naturally occurring antibiotic isolated from soil sample, and it was first introduced into clinical practice in 1963 (Macleod et al., 1964). Lincomycin and its derivatives belong to lincosamide antibiotics and exhibit biological activities against anaerobic and some protozoal infections by inhibiting protein synthesis in sensitive targets (Spizek and Rezanka, 2017). Clindamycin is a semi-synthetic chlorinated derivative of lincomycin and it is marked by being one of the 20 most important antibiotics (Spizek and Rezanka, 2004a). Given the extensive clinical application of lincomycin, multiple attempts have been taken into industrial practice to increase the production yields of lincomycin or to optimize the products (Spizek and Rezanka, 2004b; Li et al., 2007). Genetic manipulations are also adopted as complement to enhance the production of lincomycin (Pang et al., 2015; Xu et al., 2018). Though the pathway of lincomycin biosynthesis was assembled mainly within the recent 10 years (Neusser et al., 1998; Novotna et al., 2004; Sasaki et al., 2012; Lin et al., 2014; Pang et al., 2015; Zhao et al., 2015; Jiraskova et al., 2016), studies on the regulation mechanism of lincomycin biosynthesis are prompted quite slowly. Thus, various methods have limited effect on yield improvement of lincomycin.

Biosynthesis of antibiotics is controlled by elaborate regulatory mechanisms. Hormone-like signaling molecules, for example γ-butyrolactone (Takano et al., 2000; Hsiao et al., 2009; Du et al., 2011), serve as stimuli that interact with their receptor proteins to prelude the secondary metabolism (Niu et al., 2016). Global regulators and/or pleiotropic regulators then deliver these signals to CSRs which directly control the onset of antibiotic biosynthesis. In addition, researches on the secondary metabolism in *Streptomyces* is expanded to the post-transcriptional regulation. For example, BldA, a rare tRNA in *Streptomyces*, has significant importance on morphological differentiation and antibiotic biosynthesis (Hackl and Bechthold, 2015). As for lincomycin biosynthesis, only limited researches contribute to decipher the regulatory network. Lu et al. (2008) found that LmbU contributes to lincomycin biosynthesis. Hou et al. (2018a, 2019) demonstrated that LmbU, as a CSR, positively regulates lincomycin biosynthesis by controlling the transcription of *lmbA*, *lmbC*, *lmbJ*, *lmbK*, *lmbW*, and *lmbU* itself, and subsequently solved the subtle mechanism of LmbU regulon. Besides, Hou et al. (2018b) also found that BldA functions as a global regulator on both morphological differentiation and lincomycin biosynthesis at the level of translation, and genes *lmbB2*, *lmbY*, and *lmbU*, which all contain TTA rare codon, get involved in the regulon. Meng et al. (2017) revealed the regulatory network between nitrate metabolism and lincomycin biosynthesis where GlnR activates the transcription of *lmrA*, the lincomycin exporter gene. Lately, a TetR-type regulator SLCG_2919 has been identified as a repressor of lincomycin biosynthesis that controls the transcription of *lmbA*, *lmbC*, *lmbE*, *lmbG*, *lmbK*, *lmbR*, *lmbV*, and *lmbW* (Xu et al., 2019). However, to complete the regulatory network of lincomycin biosynthesis, there are lots of gaps remained.

AdpA was previously found to be an A-factor dependent regulator and repressed by ArpA (Kato et al., 2007). AdpA amplifies the A-factor signal and thus participates in morphological differentiation and secondary metabolism. By means of chromatin affinity precipitation (ChAP) and chromatin immunoprecipitation (ChIP), Higo et al. (2012) found that AdpA controls more than 500 genes in *Streptomyces griseus*. Afterward AdpA is considered to be a regulator of great importance in *Streptomyces*. In *S. chattanoogensis*, AdpA_ch_ controls the expression of *wblA*_*ch*_, and thus participates in the regulation of natamycin biosynthesis (Yu et al., 2014). In *S. roseosporus*, AdpA controls the expression of *atrA* and indirectly control daptomycin biosynthesis (Mao et al., 2015). In a recent research, AdpA interacts with the two-component system PhoRP and both of them contribute to the transcription of *atrA* (Zheng et al., 2019). Effects of AdpA on antibiotic biosynthesis is always a popular topic. AdpA always serves as an activator of antibiotic biosynthesis, a classic case is that AdpA activates the transcription of *strR*, which encodes the CSR of streptomycin biosynthesis. Therefore, AdpA regulates streptomycin biosynthesis positively and indirectly (Tomono et al., 2005). Similarly, for the biosynthesis of grixazone (Higashi et al., 2007), nikkomycin (Pan et al., 2009), and natamycin (Yu et al., 2018), AdpA activates the transcription of CSRs in their BGCs and indirectly regulates antibiotic biosynthesis. On the other hand, AdpA has a negative impact on oviedomycin biosynthesis in *S. ansochromogenes* by repressing the transcription of CSR (Xu et al., 2017). Very recently, it was reported that AdpA from *S. xiamenensis* 318 negatively regulates morphological differentiation as well as polycyclic tetramate macrolactams (PTMs) production, while positively regulates xiamenmycin production by activating the transcription of two of the structural genes *ximA* and *ximB* (Bu et al., 2019). As we can speculate from previous studies, AdpA typically controls the CSR and/or some structural genes in an antibiotic BGC. So, we scanned the lincomycin BGC and found that putative AdpA binding sites were extraordinarily scattered upstream all of the 8 putative operons with different amounts and locations. Thus, we focused on the pleiotropic regulator AdpA (GenBank accession no. ANS65440.1) and attempted to investigate its regulatory mechanism of lincomycin biosynthesis in *Streptomyces lincolnensis* in this study.

## Materials and Methods

### Bacterial Strains, Plasmids, and Culture Conditions

Bacterial strains and plasmids used in this study are listed in Table 1. *S. lincolnensis* NRRL 2936 which served as wild type (WT) and its mutants were incubated at 28°C on mannitol soya flour (MS) medium (Kieser et al., 2000) for 3–5 days for routine cultivation, phenotype observation, and strain preservation, and then cultivated at 28°C in YEME liquid medium [10 g/L yeast extract (OXOID, United States), 5 g/L polypeptone (Nihon Pharmaceutical, Japan), 10 g/L glucose (Lingfeng, China), 3 g/L maltose (Generay, China), 5 mM MgCl_2_⋅2H_2_O (Lingfeng, China), 340 g/L sucrose (Titan, China), dissolved in dH_2_O] with shaking (210 rpm) for 3–5 days for routine cultivation, total DNA extraction, and sporeless strain preservation. Fermentation medium FM1 [20 g/L lactose (SCRC, China), 20 g/L glucose, 10 g/L polypeptone, 10 g/L corn steep liquor (Aladdin, China), dissolved in dH_2_O] is used for primary cultivation, and FM2 [20 g/L lactose, 20 g/L glucose, 10 g/L polypeptone, 10 g/L corn steep liquor, 4 g/L CaCO_3_ (Lingfeng, China), dissolved in dH_2_O] is used for dry cell weight determination and lincomycin production assays. ISP4 medium [10 g/L soluble starch (Lingfeng, China), 1 g/L K_2_HPO_4_ (Lingfeng, China), 5 g/L MgSO_4_⋅7H_2_O (Lingfeng, China), 1 g/L NaCl (Titan, China), 2 g/L (NH_4_)_2_SO_4_ (Lingfeng, China), 2 g/L CaCO_3_, 15 g/L Agar (Shize, China), 0.001 g/L FeSO_4_⋅_7_H_2_O, 0.001 g/L MnCl_2_⋅4H_2_O (Lingfeng, China), 0.001 g/L ZnSO_4_⋅7H_2_O (Lingfeng, China), 0.02 mol/L MgCl_2_, dissolved in dH_2_O] was used for conjugation of *Escherichia coli* and *S. lincolnensis*. Antibiotics were supplemented on demand with the following final concentration: 20 μg/mL apramycin (Sangon Biotech, China), 20 μg/mL kanamycin (Kinglyuan, China), 12 μg/mL chloramphenicol (Sigma-Aldrich, United States), and/or 20 μg/mL nalidixic acid (Aladdin, China).

**TABLE 1:** Strains and plasmids used in this study.

**Strain or plasmid**	**Genotype and/or description**	**Source or references**
**Strains**		
***S. lincolnensis***		
NRRL 2936	Wild type (WT), lincomycin producer	NRRL, United States
Δ*adpA*	Deletion of *AdpA*_*lin*_, with an insertion of the neomycin resistance gene cassette	This study
Δ*adpA*:*adpA*	Δ*adpA* attBΦC31:pADO	This study
WT:pADPX	NRRL 2936 attBΦC31:pADPX	This study
Δ*adpA*:pADPX	Δ*adpA* attBΦC31:pADPX	This study
WT:pBLPX	NRRL 2936 attBΦC31:pBLPX	This study
Δ*adpA*:pBLPX	Δ*adpA* attBΦC31:pBLPX	This study
WT:pUPX	NRRL 2936 attBΦC31:pUPX	This study
Δ*adpA*:pUPX	Δ*adpA* attBΦC31:pUPX	This study
WT:pAPX	NRRL 2936 attBΦC31:pAPX	This study
Δ*adpA*:pAPX	Δ*adpA* attBΦC31:pAPX	This study
WT:pCPX	NRRL 2936 attBΦC31:pCPX	This study
Δ*adpA*:pCPX	Δ*adpA* attBΦC31:pCPX	This study
WT:pDPX	NRRL 2936 attBΦC31:pDPX	This study
Δ*adpA*:pDPX	Δ*adpA* attBΦC31:pDPX	This study
WT:pJPX	NRRL 2936 attBΦC31:pJPX	This study
Δ*adpA*:pJPX	Δ*adpA* attBΦC31:pJPX	This study
WT:pKPX	NRRL 2936 attBΦC31:pKPX	This study
Δ*adpA*:pKPX	Δ*adpA* attBΦC31:pKPX	This study
WT:pVPX	NRRL 2936 attBΦC31:pVPX	This study
Δ*adpA*:pVPX	Δ*adpA* attBΦC31:pVPX	This study
WT:pWPX	NRRL 2936 attBΦC31:pWPX	This study
Δ*adpA*:pWPX	Δ*adpA* attBΦC31:pWPX	This study
*E. coli*		
JM83	F’ *ara*Δ(*lac-pro* AB) *rpsL* (Str^r^)*^a^* Φ80 *lacZ*ΔM15	Our lab
BL21 (DE3)	F^–^ *ompT hsdS gal dcm*	Novagen
ET12567:pUZ8002	*dam-13*:Tn*9 dcm-6 hsdM*; contains the non-transmissible RP4 derivative plasmid pUZ8002	Our lab
*M. luteus* 28001	Indicator strain used for the bioassay method of lincomycin production	CGMCC
**Plasmids**		
pOJ260-NEO	A suicide vector in Streptomyces	Our lab (Liu et al., 2015)
pMJ1	A suicide vector in Streptomyces, derived from pOJ260-NEO	Our lab
[0.2pt] pADNU	pMJ1 with *AdpA*_*lin*_ replaced by neomycin resistance cassette	This study
pSET152	Integrative vector based on ΦC31 integrase	Our lab (Bierman et al., 1992)
pADC	pIB152 with *AdpA*_*lin*_ inserted downstream of *ermE^∗^p*	This study
pET-28a (+)	*E. coli* expression vector	Novagen
pADH	AdpA_lin_ cloned in *Nde*I/*Eco*RI sites of pET-28a (+)	This study
pADPX	pSET152 with the *xylTE* reporter gene controlled by *adpAp*	This study
pBLPX	pSET152 with the *xylTE* reporter gene controlled by *bldAp*	This study
pUPX	pSET152 with the *xylTE* reporter gene controlled by *lmbUp*	This study
pAPX	pSET152 with the *xylTE* reporter gene controlled by *lmbAp*	This study
pCPX	pSET152 with the *xylTE* reporter gene controlled by *lmbCp*	This study
pDPX	pSET152 with the *xylTE* reporter gene controlled by *lmbDp*	This study
pJPX	pSET152 with the *xylTE* reporter gene controlled by *lmbJp*	This study
pKPX	pSET152 with the *xylTE* reporter gene controlled by *lmbKp*	This study
pVPX	pSET152 with the *xylTE* reporter gene controlled by *lmbVp*	This study
pWPX	pSET152 with the *xylTE* reporter gene controlled by *lmbWp*	This study

*Escherichia coli* strains were cultivated at 37°C in Luria-Bertani (LB) liquid medium with shaking (180 rpm) or on LB solid media. Antibiotics were supplemented on demand with the following final concentration: 50 μg/mL apramycin, 50 μg/mL kanamycin, and/or 30 μg/mL chloramphenicol.

*Micrococcus luteus* 28001 were cultivated at 37°C in medium III (5 g/L polypeptone, 1.5 g/L beef extract (SCRC, China), 3 g/L yeast extract, 3.5 g/L NaCl, 3.68 g/L K_2_HPO_4_, 1.32 g/L KH_2_PO_4_ (Lingfeng, China), 1 g/L glucose, 18 g/L agar) for 16 to 18 h.

### Deletion and Complementation of *AdpA*_*lin*_

To construct *AdpA*_*lin*_ disruption strain Δ*adpA* in *S. lincolnensis*, DNA fragments of upstream and downstream region of *AdpA*_*lin*_ were amplified separately using primers *ad*-F1/R1 and *ad*-F2/R2. Then digested, respectively, with restriction enzyme *Hin*d III/*Xba* I and *Bam*HI/*Eco*R I (Thermo Fisher Scientific, United States) and ligated into the *E. coli-Streptomyces* shuttle plasmid pMJ1 to generate plasmid pADNU. Then *E. coli* ET12567/pUZ8002 was used to introduce pADNU into *S. lincolnensis* NRRL 2936 by conjugal transfer (Hou et al., 2018b). As a result of homologous recombination Δ*adpA* was constructed. DNA sequencing with primers ID*ad*-F1/IDneo-R1 and IDneo-F2/ID*ad*-R2 was adopted for further identification.

To construct *adpA* complementation strain Δ*adpA*:*adpA*, a DNA fragment covering *AdpA*_*lin*_ was amplified by PCR with primers *ad*-C-F/R and then digested with *Nde* I/*Eco*R I (Thermo Fisher Scientific, United States). Then ligated into the corresponding sites of the integrative vector pSET152. The resulting plasmid pADC was introduced into Δ*adpA* by conjugal transfer and integrated into the chromosome to generate Δ*adpA*:*adpA* where the complemented *AdpA*_*lin*_ was under the control of the promoter *ermE*^∗^*p*. DNA sequencing with primers 152-F/R was adopted for further identification.

All primers used in this study are listed in Supplementary Table S1, and synthesized by Genewiz (China).

### Scanning Electron Microscope (SEM)

Scanning electron microscope assay referred to a previously established method (Hou et al., 2018b) with some optimizations. *S. lincolnensis* NRRL 2936, Δ*adpA*, and Δ*adpA*:*adpA* were cultured on SMA medium at 28°C for about 5 days. Equivalent areas of the lawn were harvested and placed in 2.5% glutaraldehyde solution overnight. Dehydrated by vacuum freezing drying and sprayed with platinum by Gatan ALTO 1000E (Gatan, United States). Then observed with Hitachi S-3400N scanning electron microscopy (Hitachi, Japan).

### Dry Cell Weight Determination and Lincomycin Bioassay Analysis

*Streptomyces lincolnensis* NRRL 2936, Δ*adpA*, and Δ*adpA*:*adpA* were inoculated from SMA medium into FM1 at 28°C with shaking (210 rpm) for 3 days and then inoculated into FM2 and cultured for 6 days at 28°C with shaking (210 rpm). Precipitate of each sample was harvested every 24 h and dried at 55°C for 24 h. Then the weights of the dried precipitates represent the dry cell weights. Meanwhile, to analyze the bioassay of lincomycin, supernatant of each sample was harvested at the same time, and previously mentioned method (Pharmacopoeia of the People’s Republic of China [PPRC], 1990; Hou et al., 2018a) with some modifications was adopted. *M. luteus* 28001, used as indicator, was cultured on medium III at 37°C for 16–18 h and the lawn was washed off with 0.9% NaCl and suspended readily to use. Lincomycin standard solutions (4, 6, 8, 10, 12, 14, and 16 μg/mL) were used for the standard curve and internal control. Diameters of inhibition zone were linearized with the logarithmic values of the concentrations of the lincomycin standard solutions. Concentration of each sample was calculated on the basis of the standard curves. All assays in this section were performed in duplication and standard errors of the mean were calculated. The software GraphPad Prism 7.00 was used to draw the line graph of dry cell weight and histogram of lincomycin bioassay.

### RNA Extraction and Quantitative Real-Time PCR (qRT-PCR)

*Streptomyces lincolnensis* NRRL 2936, and Δ*adpA* cultured on the second day in FM2 medium were used to extract total RNA. Precipitate of samples were ground in liquid nitrogen (Liu et al., 2013) and followed by the method using TRIzol (Thermo Fisher Scientific, United States) (Setinova et al., 2017). After reacting with Recombinant DNase I (Takara, Japan) to remove the trace amount of DNA, 800 ng of RNA samples (analyzed by NanoDrop 2000, Thermo Fisher Scientific, United States) were reverse transcribed to cDNA using Reverse Transcriptase M-MLV (Takara, Japan). SYBR green PCR master mix (ToYoBo, Japan) was used and qRT-PCR was performed in triplication for each transcript. qRT-PCR conditions were mentioned previously (Hou et al., 2018a). To detect the transcript level of AdpA_lin_ targets, primers q*bl*-F/R, q*U*-F/R, q*A*-F/R, q*C*-F/R, q*D*-F/R, q*J*-F/R, q*K*-F/R, q*V*-F/R, and q*W*-F/R in Supplementary Table S1 were used. And primers q*hrdB*-F/R were used to detect the transcript level of *hrdB* which served as an internal control. qRT-PCR was performed with samples in triplication and data were treated with the threshold cycle (2^–ΔΔC^_T_) method (Livak and Schmittgen, 2001) and standard errors of the mean were calculated. GraphPad Prism 7.00 was used to draw the histogram of relative expression level of each AdpA_lin_ target.

### Catechol Dioxygenase Activity Analysis

DNA fragment covering reporter gene *xylTE* was amplified by PCR with primers *xyl*-F/R. Promoters of different AdpA_lin_ targets were amplified separately by PCR with primers *adp-xyl*-F/R (for *adpAp*, from −610 to +4), *blp-xyl*-F/R (for *bldAp*, from −799 to +52), *Up-xyl*-F/R (for *lmbUp*, from −730 to +17), *Ap-xyl*-F/R (for *lmbAp*, from −533 to +3), *Cp-xyl*-F/R (for *lmbCp*, from −513 to −1), *Dp-xyl*-F/R (for *lmbDp*, from −581 to +3), *Jp-xyl*-F/R (for *lmbJp*, from −391 to +3), *Kp-xyl*-F/R (for *lmbKp*, from −896 to +3), *Vp-xyl*-F/R (for *lmbVp*, from −364 to −1), and *Wp-xyl*-F/R (for *lmbWp*, from −456 to +4). In respect to *bldAp*, +1 represents the start point of mature *bldA*. As for other promoters, +1 represents the translation starting point of the genes controlled by them. Promoter fragment and *xylTE* fragment were inserted into Pvu II site of the integrative vector pSET152 using Super Efficiency Fast Seamless Cloning kits (DoGene, China) (Hou et al., 2018b) to construct reporter plasmids pADPX, pBLPX, pUPX, pAPX, pCPX, pDPX, pJPX, pKPX, pVPX, pWPX, and pADPX2. Then introduced into *S. lincolnensis* NRRL 2936 or Δ*adpA* respectively, to investigate the effects of AdpA_lin_ on these targets. Referred to the method optimized by Hou et al. (2018b), Catechol dioxygenase activity analysis was carried out in triplication. Standard errors of the mean were calculated and the software GraphPad Prism 7.00 was used to draw the histogram of catechol dioxygenase activity.

### Electrophoretic Mobility Shift Assays (EMSAs)

The *AdpA*_*lin*_ gene was amplified by PCR with primers *ad*-C-F/R and digested with *Nde* I/*Eco*R I. DNA fragment was cloned into corresponding sites of pET-28a (+) vector (Novagen, United States), and the resulting plasmid pADH was transformed into *E. coli* BL21 (DE3). Overexpression and purification of recombinant protein refer to the procedures described previously (Hou et al., 2018a). According to our experience, DNA probes with length around 200 bp are appropriate for EMSAs with AdpA_lin_. For the first round of amplification, primers *adp*-A-F/R, *adp*-B-F/R, *adp*-B-1-F/R, *adp*-B-F/*adp*-B-2-R, *adp*-B-F/m*adp*-B-2-R, *blp*-A-F/R, *blp*-B-F/R, *blp*-N-F/R, m*blp*-B-F/*blp*-B-R, *Up*-A-F/R, *Up*-B-F/R, *Up*-C-F/R, m*Up*-B-F/R, *Ap*-A-F/R, m*Ap*-F/R, *Cp*-A-F/R, *Cp*-B-F/R, *Cp*-C-F/R, *Dp*-A-F/R, *Jp*-A-F/R, m*Jp*-A-F/R, *Jp*-B-F/R, *Kp*-B-F/R, m*Kp*-B-F/R, and *Vp*-A-F/R in Supplementary Table S1 were used to amplify DNA probes with putative AdpA binding sites, and primers n*ad*-F/R were used to amplify DNA probe with no AdpA binding site as a negative control. Genes *lmbV* and *lmbW* share the same DNA probe. For the second round, amplified DNA fragments are used as templates with primer Biotin-linker^∗^ to harvest DNA probes with biotin labeled at 5′ terminal. EMSAs were performed as previously described (Liao et al., 2015) using chemiluminescent EMSA kits (Beyotime Biotechnology, China). AdpA_lin_ of different concentrations (0, 1.6, 3.2, and/or 6.4 μM, respectively) interacted with 2.5 nM biotin labeled DNA probe in binding buffer TGB [20 mM Tris–HCl (Shize, China), 5% glycerol (Titan, China), and 0.1% BSA (Sangon, China), pH 7.5], and 200-folds excess of unlabeled probes were added as competitive assays.

## Results

### AdpA_lin_ Positively Regulates Both Lincomycin Biosynthesis and Morphological Differentiation in *S. lincolnensis*

It has been shown that AraC/XylS family regulators control various metabolic pathways including antibiotic biosynthesis (Ibarra et al., 2008). There are about 30 AraC/XylS family regulators in *S. lincolnensis*, among which, AdpA is the most famous one. Based on this, we investigated the effects of AdpA_lin_ on lincomycin biosynthesis and attempted to propose some innovative idea on this classic regulator. Alignment of AdpA from 26 *Streptomyces* species (Supplementary Figures S1A,B) showed that AdpA retained the conserved N-terminal ThiJ/PfpI/DJ-1-like (also referenced as GATase-1) dimerization domain and C-terminal AraC/XylS-type DNA-binding domain (DBD) (Ohnishi et al., 2005). The first 340 amino acids possessed an ortholog with over 90% identities, and the main diversity occurred at the tail end of C-terminus with a length of no more than 90 amino acids, after the conserved DNA binding domain (Supplementary Figure S1A). To infer the evolutionary history of AdpA, phylogenetic analysis was performed using a maximum likelihood method. The results showed that AdpA_lin_ possessed an ortholog with 89% amino acid identity to AdpA_sg_, and thus we classified AdpA_lin_ to be one member of the AraC/XylS family.

**FIGURE 1:**
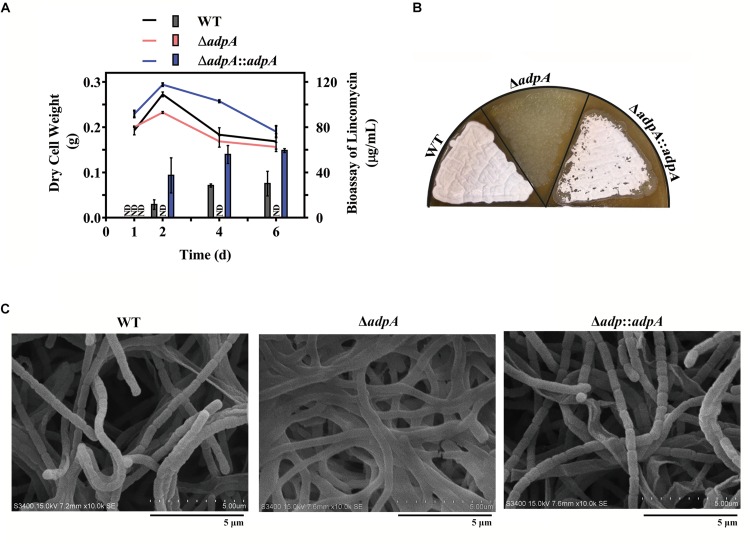
Effects of *AdpA*_*lin*_ on lincomycin biosynthesis and morphological differentiation. **(A)** Changing of dry cell weight (line graph), which represented growth curve. And bioassay of lincomycin (histogram) of WT, Δ*adpA* and Δ*adpA*:*adpA*, which showed the production of lincomycin. ND represents not detected. **(B,C)** Growth of WT and *AdpA*_*lin*_ mutants cultivated on SMA medium. **(B)** Lawns of WT and *AdpA*_*lin*_ mutants grown on SMA medium. **(C)** Scanning electron micrograph with a scale bar of 5 μm.

To investigate the effects of AdpA_lin_ on lincomycin biosynthesis, *AdpA*_*lin*_ null mutant was constructed and named as Δ*adpA*. Lincomycin biosynthesis was significantly influenced by the non-functional *AdpA*_*lin*_. In medium FM2, lincomycin started to be produced on the second day in WT, reached a maximum bioassay of 30.10 μg/mL between the second and the fourth day, and maintained thereafter. However, bioassay of lincomycin in Δ*adpA* remained undetectable throughout the entire 6 days (Figure 1A). Furthermore, the lawns of Δ*adpA* on SMA medium exhibited a bald phenotype distinct from WT (Figure 1B). Deletion of *AdpA*_*lin*_ blocked the sporulation and caused long, extended aerial hyphae when detected by SEM (Figure 1C). Complementation of *AdpA*_*lin*_ in Δ*adpA* strain (Δ*adpA*:*adpA*) restored both lincomycin biosynthesis and sporulation as expected (Figure 1) though lincomycin production in complemented strain did not restore to WT level, probably due to using the promoter *ermE^∗^p*. Moreover, the biomasses of WT, Δ*adpA*, and Δ*adpA*:*adpA* were measured at all the four detected days. The data showed that biomasses of the three strains have no significant differences at days 1 and 6, while at day 2, Δ*adpA* had decreased biomass compared to WT and Δ*adpA*:*adpA*, and at day 4, Δ*adpA*:*adpA* had increased biomass compared to WT and Δ*adpA*. These data suggested that AdpA_lin_ is an important regulator of lincomycin biosynthesis in *S. lincolnensis*.

### AdpA_lin_ Directly Activates Transcription of the Structural Genes in the *lmb* Cluster

AdpA regulates more than 500 genes in *S. griseus* (Higo et al., 2012), and AdpA binding motifs have been well studied in other *Streptomyces* species such as *S. griseus* (Yamazaki et al., 2004), *S. coelicolor* (Kim et al., 2005), and *S. lividans* (Guyet et al., 2013). Additionally, Ming et al. have solved the complex structure of AdpA-DBD and target DNA in *S. griseus* (Yao et al., 2013). AdpA binding site is recognized as 5′-TGGCSNGWWY-3′ (where S is G or C, W is A or T, Y is T or C, and N is any nucleotide), and G at position 2 and C at position 4 are more highly conserved than the other nucleotides in this motif (Yao et al., 2013). Lincomycin BGC was named as the *lmb* cluster, and the gene organization was shown in Supplementary Figure S2A. The *lmb* cluster contains 8 putative operons and the first genes of them are *lmbA*, *lmbC*, *lmbD*, *lmbJ*, *lmbK*, *lmbV*, *lmbW*, and *lmbU*, respectively. We looked into the nucleic acid sequence of the *lmb* cluster and searched with the conserved AdpA binding sequence, and no more than 3 mismatches in the last 3 bp were allowed. We found that putative AdpA binding sites were scattered in the upstream region of all the 8 putative operons (Supplementary Figures S2B–F). The numbers of putative AdpA binding sites in the upstream of *lmbA*, *lmbC*, *lmbD*, *lmbJ*, *lmbK*, *lmbV*, *lmbW*, and *lmbU* are 1, 6, 1, 3, 3, 2, 2, and 10, respectively. Therefore, we speculated that the entire biosynthesis process of lincomycin might be under the control of AdpA_lin_.

**FIGURE 2:**
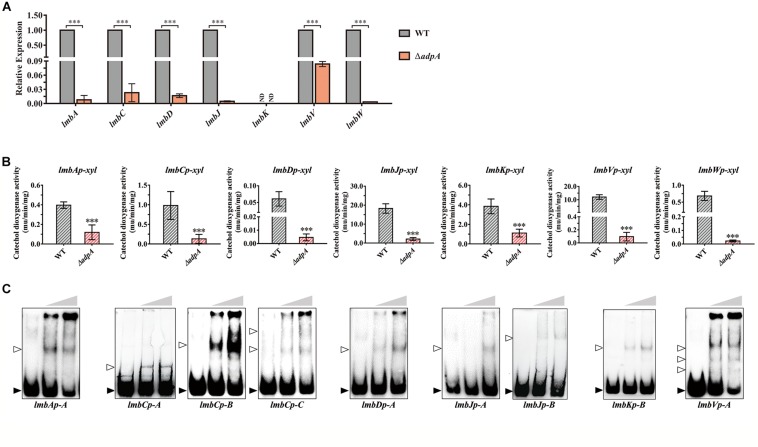
AdpA_lin_ activates the transcription of structural genes in *lmb* cluster. **(A)** Relative expression of *lmbA*, *lmbC*, *lmbD*, *lmbJ*, *lmbK*, *lmbV*, and *lmbW* in WT and Δ*adpA*. ND means not detected. **(B)** Catechol dioxygenase activity assays of WT and Δ*adpA* transformed with corresponding reporter plasmids. **(C)** EMSAs of AdpA_lin_ with 5′-biotin labeled probes in Supplementary Figure S2. Solid triangles point to the bands of probes and hollow triangles point to the complexes of AdpA_lin_ and probes. Concentrations of AdpA_lin_ are 0, 1.6, and 3.2 μM, respectively. ^∗∗∗^*P* < 0.001.

qRT-PCR analysis showed that the transcript level of *lmbA*, *lmbC*, *lmbD*, *lmbJ*, *lmbV*, and *lmbW* dramatically decreased in Δ*adpA* with fold changes 129.41, 43.64, 60.30, 215.29, 11.95, and 301.20, respectively (Figure 2A). It was suggested that in Δ*adpA*, lincomycin biosynthesis was blocked because of the decreased expression of structural genes *lmbA*, *lmbC*, *lmbD*, *lmbJ*, *lmbV*, and *lmbW*. Due to the low transcript level of *lmbK* (data not shown), we failed to calculate the relative expression in both WT and Δ*adpA*. To investigate the regulation between AdpA_lin_ and *lmbK*, we cloned the 895 bp of DNA sequences upstream from *lmbK* translation starting site (TSS) and constructed reporting plasmid for catechol dioxygenase activity assay. The results demonstrated that AdpA_lin_ got involved in regulating the transcription of *lmbK* (Figure 2B). Besides, transcriptions of *lmbA*, *lmbC*, *lmbD*, *lmbJ*, *lmbV*, and *lmbW* were activated by AdpA_lin_ in catechol dioxygenase activity assay as well (Figure 2B). These data suggested that AdpA_lin_ has primary effects on the structural genes in the *lmb* cluster.

Then we carried out EMSAs to investigate the interplay between AdpA_lin_ and promotors of above *lmb* structural genes. DNA fragments containing putative AdpA binding sites were labeled with biotin and incubated with purified AdpA_lin_. Results of EMSAs showed that AdpA_lin_ interacted with all of the 8 promoter regions containing putative AdpA binding sites (Figure 2C). DNA probe *lmbJp-A* served as part of the promoter regions of both *lmbJ* and *lmbK*, and similarly, DNA probe *lmbVp-A* served as the promoter regions of both *lmbV* and *lmbW.* For promoters containing more than one AdpA binding sites, such as genes *lmbC*, *lmbJ*, *lmbK*, *lmbV*, and *lmbW*, AdpA_lin_ interacted with different putative AdpA binding sites and generated different forms of complexes (Figure 2C). DNA fragment without AdpA binding site, i.e., probe-neg, could not interact with AdpA_lin_ (Supplementary Figure S3). To confirm the exact binding sites of AdpA_lin_ with promoters, we deleted the putative AdpA binding sites in *lmbAp*-A, *lmbJp*-A and *lmbKp*-B, and EMSAs showed AdpA_lin_ no longer interacted with these DNA probes (Supplementary Figure S4). Thus, we speculated that AdpA_lin_ activates all of the 8 promoters in the *lmb* cluster by directly binding to putative AdpA binding sites.

### AdpA_lin_ Directly Activates Transcription of the CSR Gene *lmbU*

**FIGURE 3:**
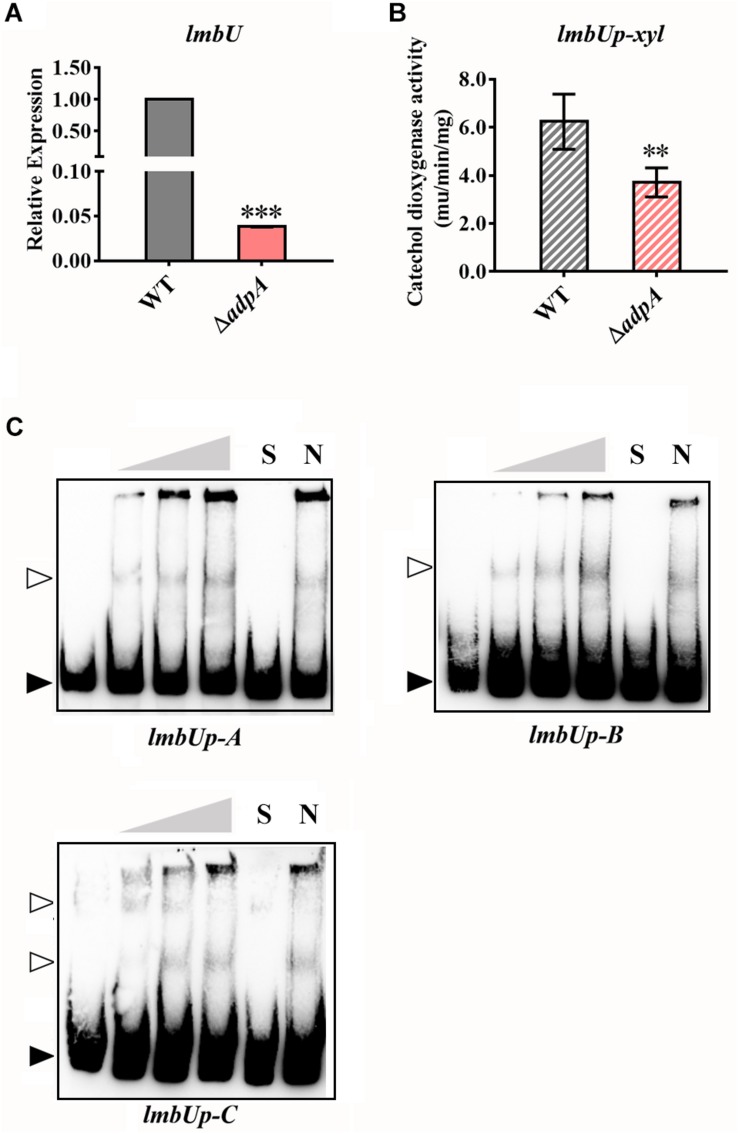
AdpA_lin_ activates the transcription of the CSR gene *lmbU*. **(A)** Relative expression of *lmbU* in WT and Δ*adpA*. **(B)** Catechol dioxygenase activity analysis of WT and Δ*adpA* transformed with pUPX. **(C)** EMSAs of AdpA_lin_ with 5′-biotin labeled DNA probes *lmbUp-A*, *lmbUp-B*, and *lmbUp-C*. Solid triangles point to the bands of probes and hollow triangles point to the complexes of AdpA_lin_ and probes. Concentrations of AdpA_lin_ are 0, 1.6, 3.2, 6.4, 6.4, and 6.4 μM, respectively. Competitive assays were carried out with a 200-fold excess of unlabeled specific probe *lmbUp*-A, *lmbUp*-B, or *lmbUp-*B (lane S) or with a 200-fold excess of unlabeled non-specific probe probe-neg (lane N). ^∗∗∗^*P* < 0.001 and ^∗∗^*P* < 0.01, respectively.

LmbU was recently reported by Hou et al. (2018a, 2019) to be a novel transcriptional regulator cited in the *lmb* cluster and positively regulate lincomycin biosynthesis. Here, we investigated the regulatory relationship between AdpA_lin_ and *lmbU*. We analyzed the 770 bp of promoter region upstream from *lmbU* TSS, and found 10 putative AdpA binding sites where two of them are overlapped (Supplementary Figure S2F). Relative expression of *lmbU* significantly decreased by 26.26 folds in Δ*adpA* compared with WT (Figure 3A). We cloned the 770 bp of DNA sequences upstream from *lmbU* TSS to construct reporter plasmid for catechol dioxygenase assay. In accordance with qRT-PCR results, catechol dioxygenase assay demonstrated that AdpA_lin_ remarkably activated *lmbU* promoter (Figure 3B). As displayed in Supplementary Figure S2F, *lmbUp-A*, *lmbUp-B*, and *lmbUp-C* are three DNA probes containing putative AdpA binding sites in the promoter region of *lmbU*. EMSAs indicated that AdpA_lin_ directly bound to *lmbUp-A*, *lmbUp-B*, and *lmbUp-C*, separately (Figure 3C). Then, the putative AdpA binding site in *lmbUp*-B was deleted, and EMSA showed that AdpA_lin_ could not bind to this DNA probe (Supplementary Figure S4), suggesting that AdpA_lin_ activates the transcription of *lmbU* by directly binding to the *lmbU* promoter and thus gets involved in the activation of lincomycin biosynthesis.

### AdpA_lin_ Directly Activates the Expression of the Global Regulator Gene *bldA*

**FIGURE 4:**
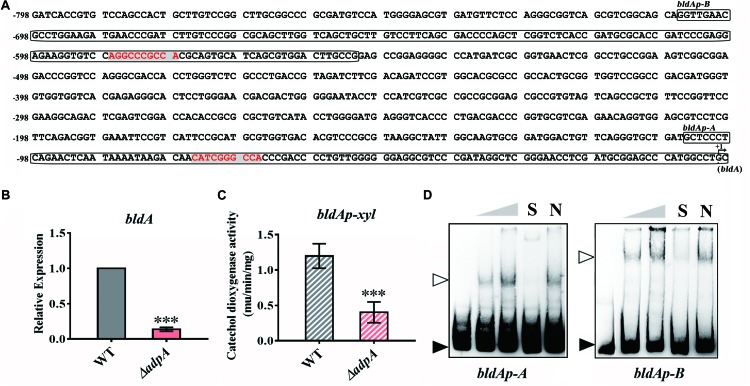
AdpA_lin_ activates the transcription of *bldA*. **(A)** Nucleic acid sequence of *bldA* promoter region where +1 represents the start of mature *bldA*. Putative AdpA binding sites are marked in red color with gray background. Probes for EMSA, *bldAp-A*, and *bldAp-B*, are framed. **(B)** Relative expression of *bldA* in WT and Δ*adpA*. **(C)** Catechol dioxygenase activity analysis of WT and Δ*adpA* transformed with pBLPX. **(D)** EMSAs of AdpA_lin_ with 5′-biotin labeled DNA probes *bldAp-A* and *bldAp-B*. Solid triangles point to the bands of probes and hollow triangles point to the complexes of AdpA_lin_ and DNA probes. Concentrations of AdpA_lin_ are 0, 1.6, 3.2, 3.2, and 3.2 μM, respectively. Competitive assays were carried out with a 200-fold excess of unlabeled specific probe *bldAp*-A or *bldAp-*B (lane S) or with a 200-fold excess of unlabeled non-specific probe probe-neg (lane N). ^∗∗∗^*P* < 0.001.

As mentioned above, deletion of *AdpA*_*lin*_ in *S. lincolnensis* not only blocked lincomycin biosynthesis, but also significantly impaired the generation of spores (Figures 1B,C), which suggested that AdpA_lin_ had complicated connections with genes outside the *lmb* cluster. Hou et al. (2018b) previously identified that BldA regulates morphological differentiation and lincomycin biosynthesis in *S. lincolnensis*. We analyzed the 799 bp of promoter region upstream from the mature *bldA*, three putative AdpA binding sites were found (Figure 4A). Relative expression of *bldA* exhibited a 7.29-fold decreased in Δ*adpA* compared with WT (Figure 4B). The 799 bp of DNA sequences upstream from the mature *bldA* was cloned to construct reporter plasmid for catechol dioxygenase activity assay. And the results indicated that AdpA_lin_ significantly activates the transcription of *bldA in vivo* (Figure 4C). *BldAp-A* and *bldAp-B* are two DNA probes containing putative AdpA binding sites marked in Figure 4A. We performed EMSAs of AdpA_lin_ with *bldAp-A* and *bldAp-B* separately, and as displayed in Figure 4D, AdpA_lin_ directly bound to DNA fragments containing putative AdpA binding sites *in vitro*. Thus, we speculated that AdpA_lin_ participates in the lincomycin biosynthesis in *S. lincolnensis* through activating the transcription of *bldA*, and indirectly mediates the morphological differentiation.

Since AdpA has been identified to regulate the transcription of *bldA* in *S. griseus* (Higo et al., 2011), we further analyzed the *bldA* promoter from *S. lincolnensis* and *S. griseus*. The data showed that putative AdpA binding site and its flanking sequence in *bldAp*-A was highly conserved (Supplementary Figure S5A), and EMSA of AdpA_lin_ with *bldAp*-A confirmed the binding (Figure 4D). However, the sequences upstream *bldAp*-A are various between the two species, which contains the AdpA binding sites in *S. griseus*, but not in *S. lincolnensis* (Supplementary Figure S5A). Further EMSA demonstrated that AdpA_lin_ cannot bind to this fragment *bldAp*-N (Supplementary Figure S5B), confirming that it is not a functional AdpA binding site in *S. lincolnensis*. Moreover, putative AdpA binding site in *bldAp*-B, which has not been studied before, was confirmed to be a AdpA binding site in *S. lincolnensis* (Supplementary Figure S5C).

### AdpA_lin_ Positively Autoregulates Its Own Transcription via Directly Binding to Its Own Promoter

**FIGURE 5:**
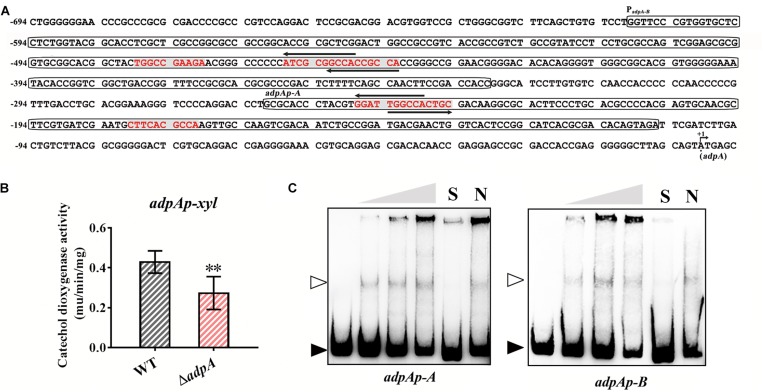
AdpA_lin_ positively regulates its own transcription. **(A)** Nucleic acid sequence of *AdpA*_*lin*_ promoter region where +1 represents the translation starting site (TSS) of *adpA*. Putative AdpA binding sites are marked in red color with gray background. Probes for EMSA, *adpAp-A* and *adpAp-B*, are framed. **(B)** Catechol dioxygenase activity analysis of WT and Δ*adpA* transformed with pADPX. ^∗∗^*P* < 0.01. **(C)** EMSAs of AdpA_lin_ with 5′-biotin labeled DNA probes *adpAp-A* and *adpAp-B*. Solid triangles point to the bands of probes and hollow triangles point to the complexes of AdpA_lin_ and DNA fragments. Concentrations of AdpA_lin_ are 0, 1.6, 3.2, 6.4, 6.4, and 6.4 μM, respectively. Competitive assays were carried out with a 200-fold excess of unlabeled specific probe *adpAp*-A or *adpAp-*B (lane S) or with a 200-fold excess of unlabeled non-specific probe probe-neg (lane N).

We carried out a detailed analysis of 610 bp of upstream DNA sequences from *AdpA*_*lin*_ TSS and found 6 putative AdpA_lin_ binding sites where 4 of them are overlapped in pairs (Figure 5A). To investigate the transcriptional regulation between AdpA_lin_ and its own transcription, we cloned the 610 bp of DNA sequence upstream from *AdpA*_*lin*_ TSS to construct a reporter plasmid for catechol dioxygenase activity assay. The results revealed that AdpA_lin_ slightly activated its own transcription *in vivo* (Figure 5B). As displayed in Figure 5A, *adpAp-A* and *adpAp-B* are two DNA probes containing putative AdpA_lin_ binding sites, and the results of EMSAs verified that AdpA_lin_ directly interacted with the promoter region of *AdpA_*lin*_ in vitro* (Figure 5C).

AdpA also has been identified to autoregulate its own transcription in *S. griseus* (Kato et al., 2005), then we compared *AdpA*_*lin*_ promoter from *S. lincolnensis* with *adpA* promoter from *S. griseus*. In *S. lincolnensis*, the putative AdpA binding site and its flanking sequence in *adpAp*-A was high conserved (Supplementary Figure S6A) and the result of EMSA verified the binding (Figure 5C). However, the nucleic acid sequences upstream *adpAp*-A were highly diverse (Supplementary Figure S6A). Then we designed two probes, *adpAp-*B-1 and *adpAp-*B-2, which contain the unconserved AdpA binding motifs from *S. griseus* (Kato et al., 2005) and putative AdpA binding sites from *S. lincolnensis*, respectively. The results of EMSAs showed that AdpA_lin_ can bind to *adpAp-*B-2, but not to *adpAp-*B-1 (Supplementary Figures S6B,C), indicating that two new AdpA binding sites are found in *S. lincolnensis*, and the differences between the two probes may allow them to respond to different regulatory mechanisms.

## Discussion

In this study, we elucidated the effect of AdpA_lin_ on lincomycin biosynthesis and morphological differentiation. There are 8 putative operons in the *lmb* cluster. Before this study, none of a regulator has been identified to directly bind to all the eight promoters in the *lmb* cluster and have an overall impact on the entire lincomycin biosynthesis progress. By deciphering the regulations on the 8 putative operons, we attempted to envision the transcriptional regulatory network on lincomycin biosynthesis. Lincomycin biosynthesis contains three main parts: formation of α-methylthiolincosaminide (MTL), formation of propylproline (PPL), and condensation and final methylation (Supplementary Figure S2A). AdpA_lin_ activates *lmbKp* and *lmbVp*, therefore we speculate that AdpA_lin_ directly activates the transcription of *lmbK*, *lmbR*, *lmbO*, and *lmbN*, so that AdpA_lin_ positively regulates the biosynthesis of MTL structure (Sasaki et al., 2012; Lin et al., 2014). AdpA_lin_ activates the transcription of *lmbAp*, *lmbWp*, and *lmbUp*, which means *lmbB1*, *lmbB2*, *lmbX* and *lmbY* are also activated by AdpA_lin_, thus we speculated that AdpA_lin_ directly regulates the biosynthesis of PPL structure (Novotna et al., 2004, 2013; Pang et al., 2015; Jiraskova et al., 2016). In addition, AdpA_lin_ activates the transcription *lmbCp*, *lmbDp*, *lmbJp*, and *lmbVp*, which means transcriptions of *lmbT*, *lmbE*, *lmbF*, and *lmbG* are activated by AdpA_lin_ (Hola et al., 2003; Zhao et al., 2015; Kadlcik et al., 2017; Zhong et al., 2017; Zhang et al., 2018). Therefore, we inferred that AdpA_lin_ functions as a primary activator of lincomycin biosynthesis and regulates the entire biosynthetic process. And this is the first case that AdpA directly activates the transcription of the overall structural genes in such a complicated antibiotic biosynthetic gene cluster (BGC). In addition, EMSAs of AdpA_lin_ with targets showed that AdpA_lin_ binds to different binding sites with different affinities (Figure 2C). Based on these results, some strategies of genetic manipulations may be proposed for hyper-production of lincomycin, such as mutation, deletion or addition of AdpA binding sites in the promoter regions of target genes.

**FIGURE 6:**
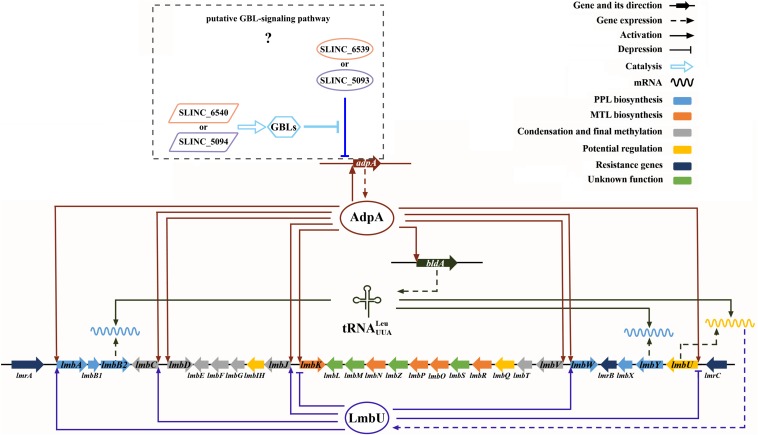
Cascade regulation of AdpA_lin_, LmbU, and BldA on lincomycin biosynthesis in *S. lincolnensis*.

Besides directly participating in lincomycin biosynthesis, as a pleiotropic regulator, AdpA_lin_ controls lincomycin biosynthesis by regulating other transcriptional regulators as well. LmbU has been identified to activate the transcription of *lmbA*, *lmbC*, and *lmbJ*, and repress the transcription of *lmbK* and *lmbU* itself (Hou et al., 2018a). In this study, we confirmed that the transcription of *lmbU* was activated by AdpA_lin_ (Figure 3). As described by Hou et al. (2018b) there is a UUA codon in *lmbU*, and translation of *lmbU* is controlled by BldA. Existence of rare codon means very small changes of the tRNA could induce the significant change of protein amount (Chater, 2006). Besides, UUA codons also exist in *lmbB2* and *lmbY* in the *lmb* cluster, indicating that LmbB2 and LmbY might be important regulatory targets during lincomycin biosynthesis. Furthermore, it has been showed that the *adpA* gene contains a UUA codon as well, on the other hand, transcription of *bldA* is regulated by AdpA_lin_ (Figure 4), which may function as a feedback regulatory mechanism to keep the organism in balance. In this study, we speculated that AdpA_lin_, LmbU, and BldA formed a regulatory cascade that mediate lincomycin biosynthesis in *S. lincolnensis* (Figure 6). In addition, considering AdpA responds to the GBL-involved cascade regulation (Healy et al., 2009; Tan et al., 2015), bioinformatics analysis was performed and two GBL-signaling systems were found in *S. lincolnensis* (Figure 6). One system consists of the GBL receptor SLINC_6539 (GenBank accession number ANS68763.1) and biosynthetic enzyme SLINC_6540 (GenBank accession number ANS68764.1) which were highly homology with many receptors and enzymes in *Streptomyces*, whereas SLINC_6539 and SLINC_6540 had identities with ArpA (47%) and AsfA (70%) in *S. griseus*, respectively. The other system consists of SLINC_5093 (GenBank accession number WP_067437987.1) and SLINC_5094 (GenBank accession number WP_067437989.1) which were low similarities with other receptors and enzymes, while SLINC_5093 and SLINC_5094 had identities with ArpA (44%) and AsfA (33%) in *S. griseus*, respectively. But how these two GBL-signaling systems works to affect lincomycin biosynthesis will be needed further research.

In *S. griseus*, AdpA auto-depresses its own transcription (Ohnishi et al., 2005), whereas in *S. lincolnensis*, AdpA_lin_ has a positive impact on its own transcription (Figure 5B). Differences of AdpA binding sites in *S. lincolnensis* and *S. griseus* indicated that AdpA homologs from different resource have specialized regulatory mechanism on their own transcription. Catechol dioxygenase activity showed that AdpA_lin_ had a negatively effect on the AdpA binding site near the *AdpA*_*lin*_ TSS (Data not shown). Thus, we speculated that AdpA_lin_ bound to this putative site and prevented the *AdpA*_*lin*_ promotor from being activated. As for another putative AdpA binding sites, we presumed it might recruit RNA polymerase after interacting with AdpA_lin_ and thus the overall effect of AdpA_lin_ on its own promoter appeared to be positive. In the natamycin producer *S. chattanoogensis*, AdpA_ch_ was an activator of natamycin biosynthesis, and 6 AdpA binding sites were identified in the *scnRI-scnRII* intergenic region (Du et al., 2011; Yu et al., 2018). It is notable that although the general effect of AdpA_ch_ on the transcription of *scnRI* is positive, AdpA binding site A and B serve as repression sites. Thus, we speculated that the varying amounts and locations of AdpA binding sites in the promoter region of AdpA_lin_ targets exhibited different effects and constituted a complicated and subtle regulatory network of AdpA regulons.

In summary, we reported a lesser-known case that AdpA_lin_ interacted with all of the 8 putative operons and activated the transcription of structural genes in the *lmb* cluster. Furthermore, we deduced AdpA_lin_, LmbU, and BldA in cascade regulation that controlled lincomycin biosynthesis. Based on these knowledge, more efforts should be devoted to complete the regulatory mechanism of lincomycin biosynthesis and to enhance to production of lincomycin.

## Data Availability Statement

All datasets generated for this study are included in the manuscript/Supplementary Files.

## Author Contributions

YK, BH, and HW designed the experiments. YK, YW, and RW carried out the experiments. YK, BH, XZ, JY, and HW analyzed the data. YK, JY, and HW wrote the manuscript. JY and HZ guided the work. All authors assisted with critical reading of the manuscript.

## Conflict of Interest

The authors declare that the research was conducted in the absence of any commercial or financial relationships that could be construed as a potential conflict of interest.
