# Supplementary material for: AdpAlin, a Pleiotropic Transcriptional Regulator, Is Involved in the Cascade Regulation of Lincomycin Biosynthesis in Streptomyces lincolnensis
Source: Front Microbiol. 2019 Oct 23;10:2428. doi: 10.3389/fmicb.2019.02428 (PMC6819324; doi:10.3389/fmicb.2019.02428)
Supplement: Supplementary file 1 [file Data_Sheet_1.PDF]

Supplementary Material

Contents:

1. Supplementary Figure S1. *In silico* analysis of AdpA from 26 *Streptomyces*

2. Supplementary Figure S2. Putative AdpA binding sites in the *lmb* cluster.

3. Supplementary Figure S3. EMSA of $AdpA_{lin}$ with a 5'- biotin labeled DNA probe that contains no $AdpA_{lin}$ binding site (probe-neg).

4. Supplementary Figure S4. EMSA of $AdpA_{lin}$ with mutated probes.

5. Supplementary Figure S5. Verification of AdpA binding sites in *bldA* promoter in *S. lincolnensis*.

6. Supplementary Figure S6. Verification of AdpA binding sites in *adpA* promoter in *S. lincolnensis*.

7. Supplementary Table S1. Primers used in this study

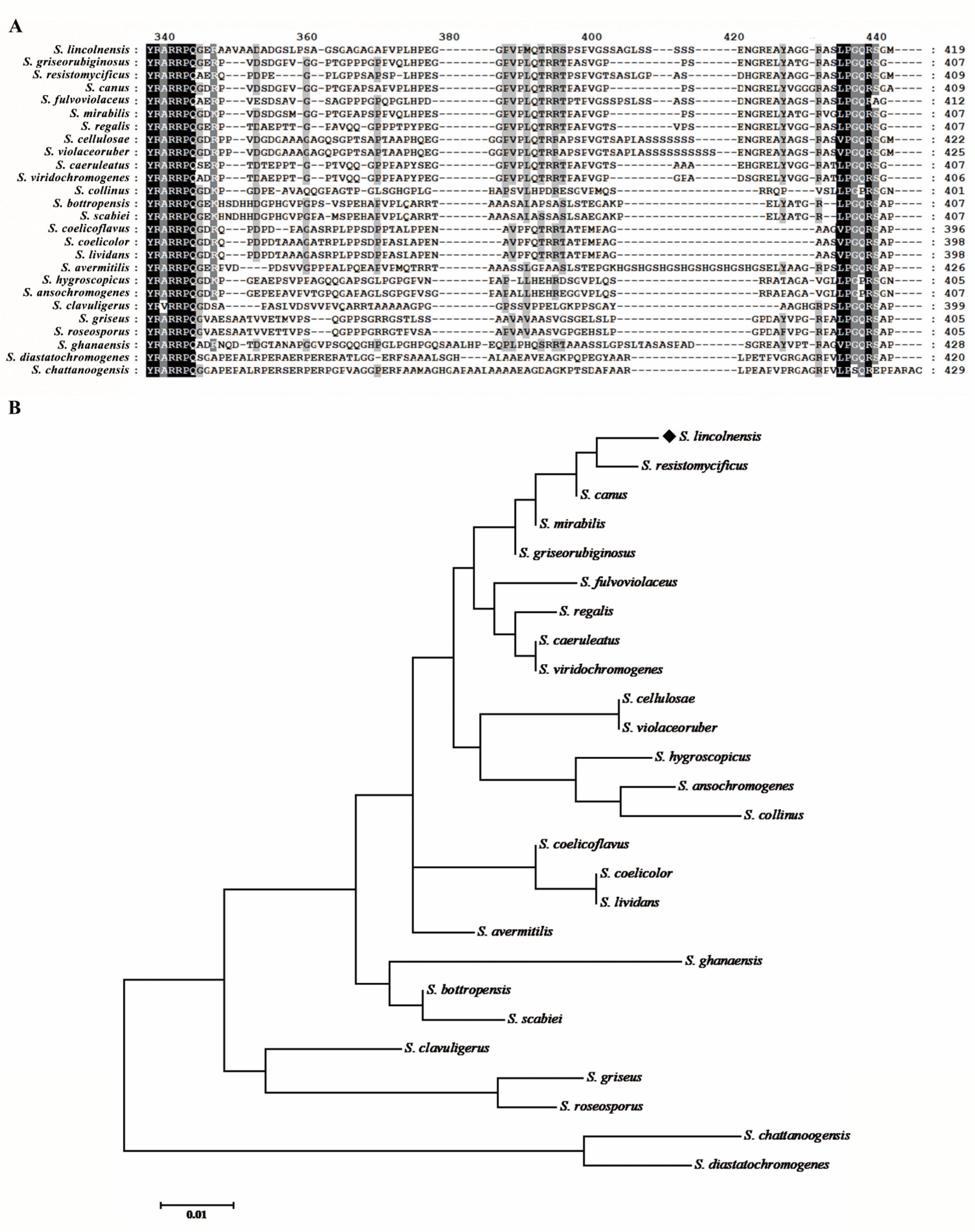
A
S. lincolnensis
S. griseorubiginosus
S. resistomycificus
S. canus
S. fulvoviolaceus
S. mirabilis
S. regalis
S. cellulosae
S. violaceoruber
S. caeruleatus
S. viridochromogenes
S. collinus
S. bottropensis
S. scabiei
S. coelicoflavus
S. coelicolor
S. lividans
S. avermitilis
S. hygroscopicus
S. ansochromogenes
S. clavuligerus
S. griseus
S. roseosporus
S. ghanaensis
S. diastatochromogenes
S. chattanoogensis
B
S. lincolnensis
S. resistomycificus
S. canus
S. mirabilis
S. griseorubiginosus
S. fulvoviolaceus
S. regalis
S. caeruleatus
S. viridochromogenes
S. cellulosae
S. violaceoruber
S. hygroscopicus
S. ansochromogenes
S. collinus
S. coelicoflavus
S. coelicolor
S. lividans
S. avermitilis
S. ghanaensis
S. bottropensis
S. scabiei
S. clavuligerus
S. griseus
S. roseosporus
S. chattanoogensis
S. diastatochromogenes
0.01

Supplementary Figure 1. *In silico* analysis of AdpA from 26 *Streptomyces* (A) Amino acid sequence comparison of AdpA. The last 100 amino acid sequences are displayed. (B) Phylogenetic analysis of AdpA. The evolutionary history of the AdpA was inferred by MEGA7 using the maximum likelihood method. GenBank accession numbers are: ANS65440.1 (*S. lincolnensis*), KUN60558.1 (*S. griseorubiginosus*), KUN99400.1 (*S. resistomycificus*), KUN72461.1 (*S. canus*), WP_030601158.1 (*S. fulvoviolaceus*), WP_037721862.1 (*S. mirabilis*), WP_062703138.1 (*S. regalis*), WP_030669749.1 (*S. cellulosae*), WP_030934456.1 (*S. violaceoruber*), KUO06406.1 (*S. caeruleatus*), EFL33074.1 (*S. viridochromogenes*), AGS69765.1 (*S. collinus*), EMF50438.1 (*S. bottropensis*), CBG72808.1 (*S. scabiei*), EHN73017.1 (*S. coelicoflavus*), NP_627022.1 (*S. coelicolor*), ACJ04048.1 (*S. lividans*), GDY75845.1 (*S. avermitilis*), AGF63724.1 (*S. hygroscopicus*), ABY86620.1 (*S. ansochromogenes*), EDY52473.1 (*S. clavuligerus*), BAA86265.1 (*S. griseus*), AIR95874.1 (*S. roseosporus*), EFE68835.1 (*S. ghanaensis*), OXY96937.1 (*S. diastatochromogenes*), and WP_053924277.1 (*S. chattanoogensis*).

A

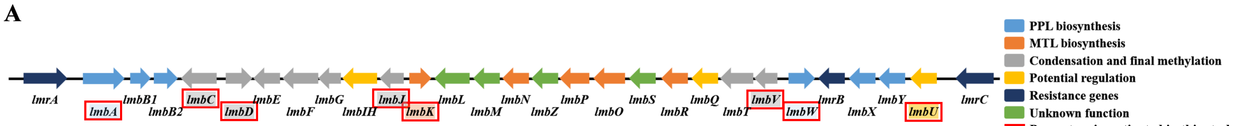

B

lmbAp-A

1 CCAGACGCTT GCCCCGACCG CGGATTACCG GTTCCCTATT CCCTATTCCC AGGTAATGCA CCGGATATCG AGGGCCGTGG CGGCACGTTC GCGCCGACTC

101 CCGGATAACT TCCAGGAATT ACGGGAAGCC GTCTGCTGCC GCTTGACAGG GCGTGAAGCC GATTGCTTGT CTCGCCGGCG AGACAGTCGG CTCCGGCATG

201 AAATCCCGTC GGCCCGGGCC CGGCTTACGG TTCCCATTGA CCTTCCCGAC AACACAGTTA TGGTGGTGGA CGCATGCTTC CATCGAAGCC GGAGCTCAGT
(*lmbA*)

C

lmbDp-A

1 GCGACGGCGC CCGCACCGGT GTGGGCCACG CACTTGGGAA CGCCGGTGGA GCCGGAGGTG GTGAGCAGGT ACGCGACATC GGTGCCGTCG CTCCGGACGG

101 GGTCGAGGCG GCCCGCGGGC AGCTCGTCCC CGTCCGATGG CAGGGCGAGC CGCCGCGGCC CCGGTACGGC GGCGGCGAAC CGGTCGAGCA GCGCGCGTTC

201 GCCCACCACG GCGGTGGGCG CGCAGACCCG GCTCACCGAA GCCCACCGCG CGGGCGGCGA CGCGGCGTCG AGCGGCACGT ACACGGCGCC CGCGGCCAGG

301 ATCGCGTGCA CCGCGGCCAC GGCGTCCGCG TCGGTCCGCC CGGACAGCAG CGCTACCCGG TCACCGGCGC GCACCCCCAG GCCTGCCAGG GCGGCCTGGA

401 TGCCGCCCGC CCGCGCGGCC AGCTCCCCAT AGGTGACCGG GAGCCGGGTC TCCCCCAGGA CGGCGGGGGC CTCGGGCCTC GCCTCGGCGG CCGAGGCGAG

(*lmbC*) *lmbCp-A*

501 GAGTGTGGAG AGTCGAACGG AGGACGACAT GAAGGACGTC GAAGAGGTCA CAGCGGCGGA CATGGCGGTA TCACCCCAGT CGGTTCGGTT GCCGGAACAC
(*lmbD*)

601 GCGCCGCACT GCGAGATCGG CTGCGCGGCG AACGTGGCGA AGAGGGTCGG TGTCGACTTC CCGCGGCACG TGGCCGGGTC CCAGTGGGCG CTGCGCACCT

lmbCp-B

701 TCGTCCGGGA GCCCGGCGAC ATCCCGCAGC CCATGCTGGA CCGCACGCAT CTGACGTTCG GCGCGCACGG CGAGGGCTGG CCCACCGCCC TCGCGGGGAT

lmbCp-C

801 CACGGACGGC ACCGTGCACG ACCGCCGGGT GGCGCGTTCG GAGCTCCCGG AGGTGCTGCG GGACGACCTC GCCCGGGGCG GCAGTCTGCT GTTCCTGGAG

901 GACCGCGGCT GCCCATGGCT GCACTCCGCC GGCCCCGGGC TGCTGCCGCA CACGGTGACC CCGGACGGTG TCGACGCGGC CGGGGTCTGG CAGCTGATCG

1001 AGGGGCACAG CTGGTGGGCG GGCCGGTACC CGATGGCGGA GGCCGACCTG CTGGCCGCGG CCTACCCGGA CCCCGACCCG CACCATGTGG CGGGCCGGGT

D

lmbKp-B

1 TCACCGTGCG CCCGGGCGTA GACGGACACG TGGTAGCGGC TGTCCGGGCG GTAGGCGGCA CCGTCGAAGT CGGCGTGGCG GCTGCGGAGT TCGAAGCCCG

101 CGAAGCGGGC CATCAGGTCC AGCTCCGTGG GGTGGGAGTA GCGCAGTGTG TACGGGAAGA AGCGGGCGCC GGACTCGCTC AGGACGATGT GATGGGCCGT

201 CAGCGTCTGA GCGACGGGAT CGTGTTTGGA GACGTCGAGG TGGACGCCGT CGTGCTCCAG CTCGACCAAC GCGACGTCAC TGCCGTCGGG CAGGGACTTG

301 GGGTTGAGGC ACTGAAGGAC CAGGAGGCCC TCCGTTTCGA GGCAGTTGGC CGCCGACCGC AGGCAGGCGA TCTGCTCGTC CTGCGTCAGC AGGCAAAAAA

401 GCGTGTTGAA CAGGGCGAAG ACGAGGCCGA AGCGGCGCTC CCCCAGATCG AGGCGGCCGA AGTCCTGTTG GTGGCCGTGC ACCGCACCGC CGCTCTTCTC

501 CTTGAGGATC TCCAGCATCC GCGCCGAGCT GTCCACGCCG TGCACCTCGA CGCCGGACTC GGCGAGCGGC ACGGCGACCC GGCCGGTACC CACGCCGAGT

lmbJp-A

601 TCGAGGGCCG GGCGGCCGTT GGCGAGTGCG GCCAGGAAGG GGGCCGCGCG GTCCGCGTCC GGGGGCGGTC CCGCGTCCCC GGGCCACACG TCGTAGACAT

(*lmbJ*)

701 CTGCGATCTT GTCACCGTAG AGCTTCGCGA AATCATGGTC GGACATTGAA TTCTCTTTCC TCACCAGGAC GTTGGCCACT CGGCGAGCGG CCGCAGGGAG

lmbJp-B

801 ACGGTGGCAT GGGACGCGAG GGACAGTCGA CACGGCAGCC GGTACCGGCG GTGTTCTTCG ACCGGGACGG CGTGCTGATC GAGGCGACGG TGCGCGACGG
(*lmbK*)

901 CAGGCCCTAC CCGCCGCGCA GTGCGGACGA CGTCCGGCTC GATCCGCACG CCGTGGACGC GGTGCTCTCC CTGCGCGCGG CCGGTTTCGC CCTGGCCGTC

1001 GTCACCAATC AGCCGGACGT GGCCCGCGGC ACGGCCACCG CGGAGGCGGT GCGCGCCGTC AACGCGCGCA CCGCCGAACT GCTCGGCATC GCCCCGGAGT

E

lmbVp-A (*lmbV*)

1 CGCAGGTGTC TTCCCTCGTC GGCCAGTGCC GTGACACCCG ACCCGAGCCG GGCCAGTCCC TTGCGCTGCA ACTCGATGGC GTAAATGGCT CTCTCCCAGC

101 TTCCGGGTGT ACGACGCGGA AGGGCGTCAG AGGTCATCAG AAGGGCGTCA GAAGGGGCCC GAGGCGGGCC AGGAAAGCAT TTGACGAGGC GGTGGGCGAT

201 CCTTCGCGAA CCGCAAGGGA AAGGTACCCG CCGGATAACG CGAAAACCAT CGCACGGAAC TGTTCTCGAC TATCCGTCGG GGCCGCAACT ACGGTGAACG

301 AGCGCAGATT TCGCCCCTGT CTCGCCGGCG GGACAATCAC AGCTTCCACG ATTGCGGCAA ACAAGCCCTT CAGACGGACC GAGGTGCCAT GACAGCCGTT
(*lmbW*)

F

lmbUp-A

1 GTCCGCAACC GATAAATTCG ACCTCACCAC GCGGCTGCCA TCCCTTTCTC ACGCTCTTGC CCTTTGGGTC ACCGAGCAGG ACACGGGGAT CGAAAGCAAG
(*lmbU*)

101 CAGCGATTCA CCCCCGTTGT TCGGCGCCCA CCACGCTCAT CGATGTCGCG CCACCGCCAC GAAGGGCGGC GCAGGCGAGC AGCTCATGAA TGGCTTGGCG

lmbUp-B

201 GAACCAGCAT CATTGCCCCC CCTCGGAACT CCGATACATG TAACGGCCAA TGTATCAAGT ACGGATCAGA GTGTAGGAGG CCGGTCAATT AATTATCCAG

301 GGGATCCTGG AGATCTTGAA ACCGGCCGGA TCCGGACCAT CCAAAGCGGC AACCCAACGG CCAAACAGCT GGGCTCATTT CATTCCGCAA GGTGAAATGA

401 GCATATGTCA ACGGCCGATT AACACGCAAG ACGCCCTCCT TTCGGAGCGA CGAATCTTTA GAGCATGACG TTCCCTGCCT TCCGCTACGT TTCTCACTTA

lmbUp-C

501 TGCCTGGATG TGTGCATTTG GGGCAGTACT GTGGAAGTTT TTAACCTTGT TTGAGAGCCC GGGCGTTTAC CTAGAACAAA TCCGGGGCAA GCTCGCTCAG

601 CTGTCGCCAT GGGCGCCCTG ACGCCACTGC GGCCCACGGA TCGCCCCGTC AGCAAACTGG CCGAATGCCA ACGGCATACG GCCAGTTCCT GACGCGGGCC

701 ACGGGGTTCA CTCCGCGGAC CACGCGTGCG GCGGCCGCCT CACTTATCCC GATGAGGCCA CGCGTCGTCC GCCTCCGCGA TCTCCTCCAG GGTTCCGTCC

Supplementary Figure 2. Putative AdpA binding sites in the *lmb* cluster. (A) Schematic diagram of the *lmb* cluster in *S. lincolnensis*. The *lmb* cluster contains 29 genes, and 8 of them were marked with colored background and analyzed in this study. (B-F) Nucleic acid sequence of promoter regions in the *lmb* cluster. Arrows show the translation start sites (TSSs) and their directions. Putative AdpA binding sites are marked in red color with grey background. The first bases of each line are numbered. DNA fragments used in EMSA were framed and named. (B) Nucleic acid sequence of upstream of *lmbA* gene. (C) Nucleic acid sequence of upstream of *lmbC* and *lmbD* genes. (D) Nucleic acid sequence of upstream of *lmbJ* and *lmbK* genes. (E) Nucleic acid sequence of upstream of *lmbV* and *lmbW* genes. (F) Nucleic acid sequence of upstream of *lmbU* gene.

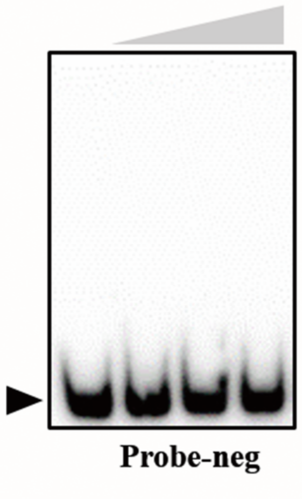

Supplementary Figure 3. EMSA of $AdpA_{lin}$ with a 5'- biotin labeled DNA probe that contains no $AdpA_{lin}$ binding site (probe-neg). Solid triangles point to the bands of DNA probes. Concentrations of $AdpA_{lin}$ are 0, 1.6, 3.2, 6.4, and 12.8 μM, respectively.

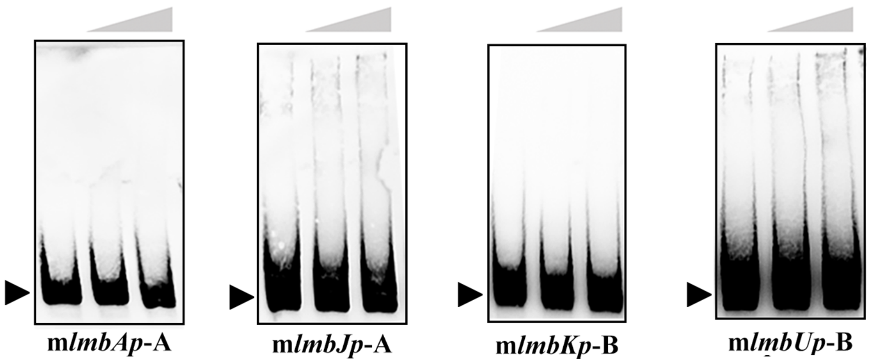

Supplementary Figure 4. EMSA of AdpA$_{lin}$ with mutated probes. Putative AdpA binding sites in *lmbAp*-A, *lmbJp*-A, *lmbKp*-B, and *lmbUp*-B were deleted and generate m*lmbAp*-A, m*lmbJp*-A, m*lmbKp*-B, and m*lmbUp*-B, respectively. For each probe, concentrations of AdpA$_{lin}$ are 0, 1.6, and 3.2 μM, respectively.

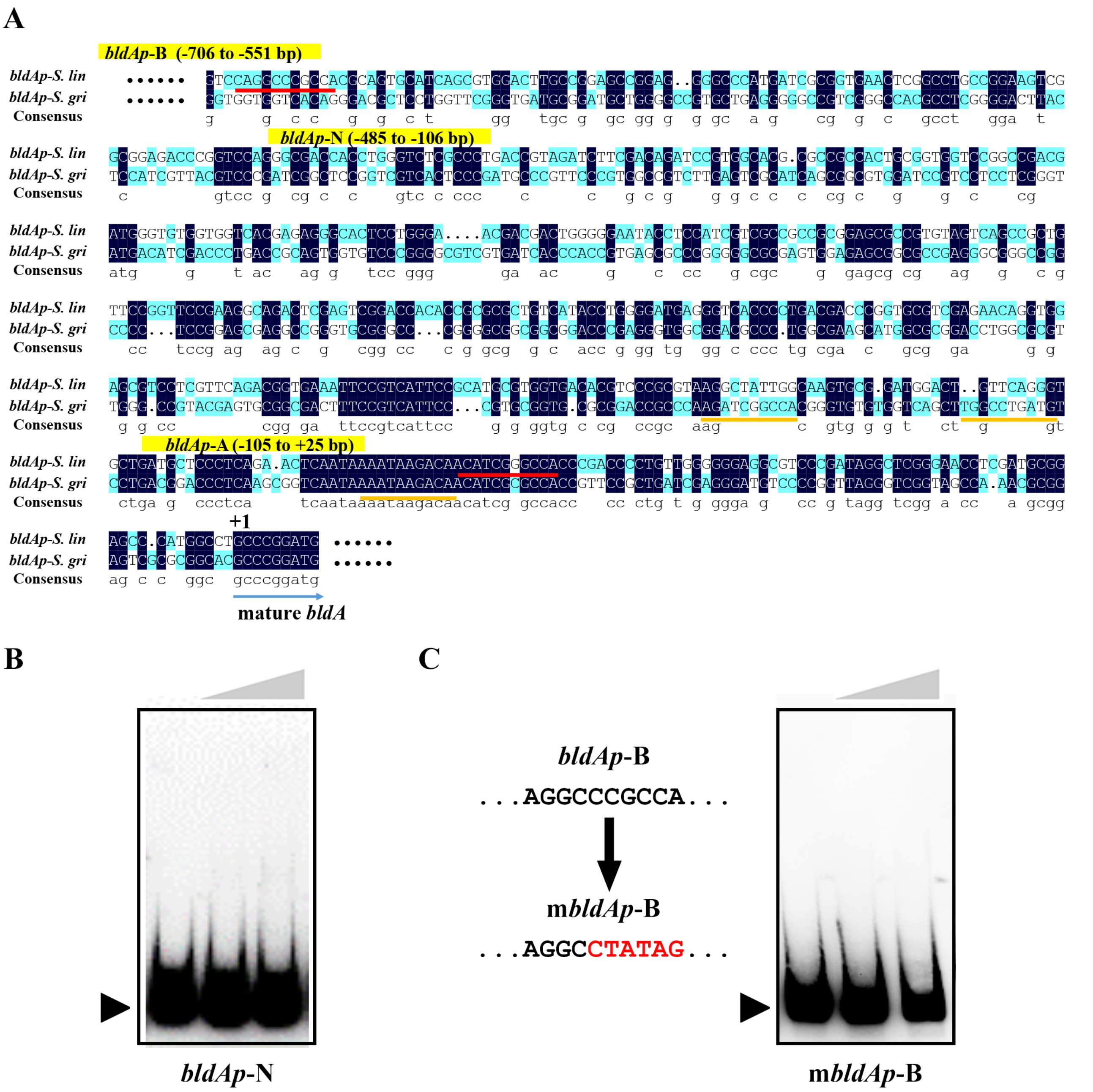

Supplementary Figure 5. Verification of AdpA binding sites in *bldA* promoter in *S. lincolnensis*. (A) Comparison of *bldA* promoter from *S. lincolnensis* and *S. griseus*. Red box represents the putative AdpA binding site in *S. lincolnensis* in this study, and yellow box represents AdpA binding site verified in the research of AdpA from *S. griseus*. +1 means the start of mature *bldA*. (B) EMSA of $AdpA_{lin}$ with DNA probe *bldAp*-N, which only contains AdpA binding site verified in the research of AdpA from *S. griseus*. Concentrations of $AdpA_{lin}$ are 0, 1.6, and 3.2 µM, respectively. (C) EMSA of $AdpA_{lin}$ with DNA probe m*bldAp*-B. Putative AdpA binding site in *bldAp*-A was mutated as shown in red letters in m*bldAp*-B. Concentrations of $AdpA_{lin}$ are 0, 1.6, and 3.2 µM, respectively.

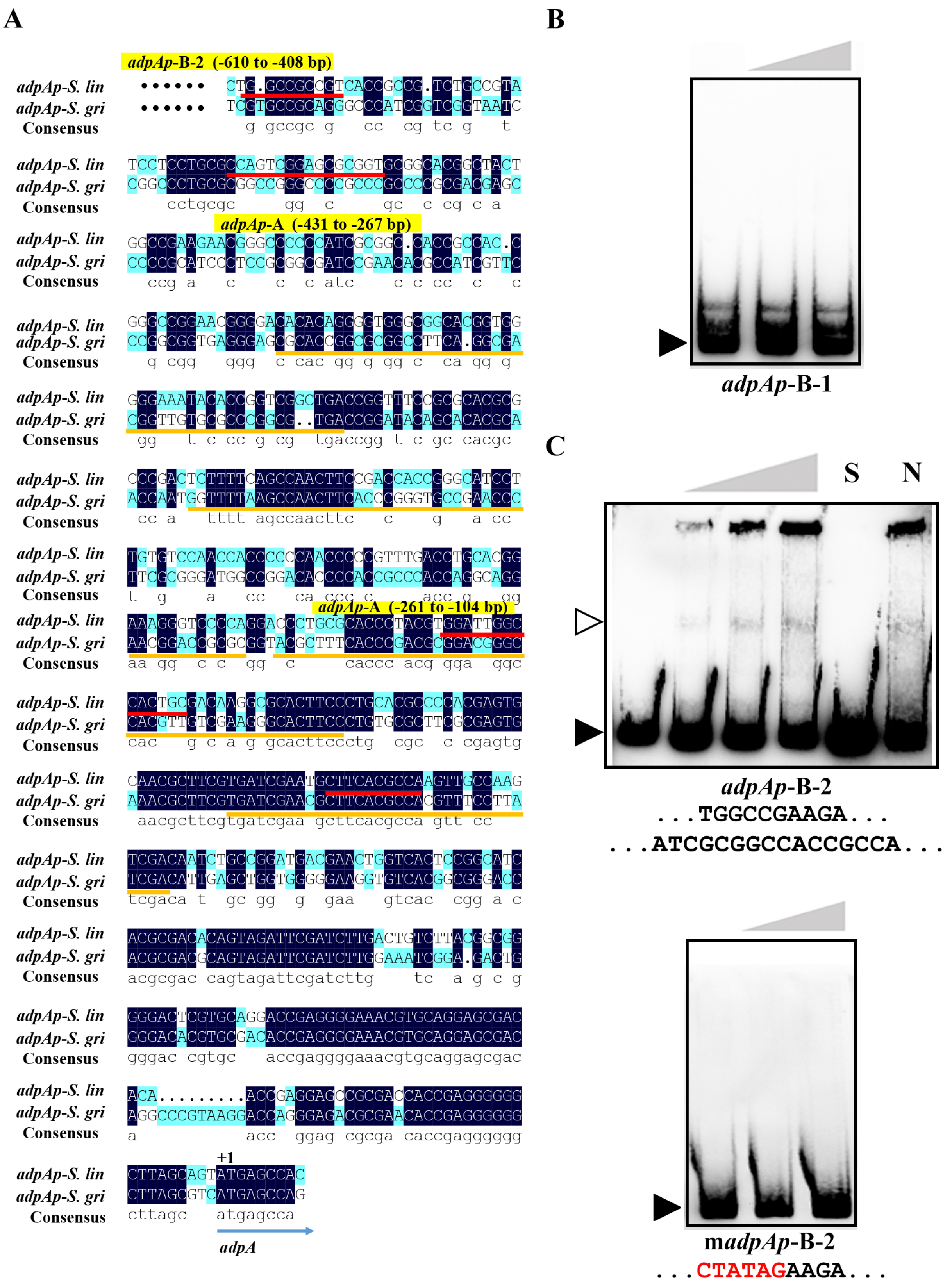
A
B
C
adpAp-B-2 (-610 to -408 bp)
adpAp-A (-431 to -267 bp)
adpAp-A (-261 to -104 bp)
adpAp-S. lin
adpAp-S. gri
Consensus
+1
adpA
adpAp-B-1
S
N
adpAp-B-2
...TGGCCGAAGA...
...ATCGCGGCCACCGCCA...
madpAp-B-2
...CTATAGAAGA...
...ATCGGATATCGGCCCCG...

Supplementary Figure 6. Verification of AdpA binding sites in *adpA* promoter in *S. lincolnensis*. (A) Comparison of *adpA* promoter from *S. lincolnensis* and *S.griseus*. Red box represents the putative AdpA binding site in *S. lincolnensis* in this study, and yellow box represents AdpA binding site verified in the research of AdpA from *S. griseus.* +1 means translation starting site of *adpA*. DNA probe *adpAp*-B was divided into two segments, *adpAp*-B-1 and *adpAp*-B-2. (B) EMSA of $AdpA_{lin}$ with *adpAp*-B-1, which only contains AdpA binding sites verified in the research of *S. griseus*. Concentrations of $AdpA_{lin}$ are 0, 1.6, and 3.2 μM, respectively. (C) EMSA of $AdpA_{lin}$ with *adpAp*-B-2 and its mutation m*adpAp*-B-2 where putative AdpA binding sites were mutated as shown in red letters. For EMSA of $AdpA_{lin}$ with *adpAp*-B-2, concentrations of $AdpA_{lin}$ are 0, 1.6, 3.2, 6.4, 6.4, and 6.4 μM, respectively. And competitive assays were carried out with a 200-fold excess of unlabeled specific probe *adpAp*-B-2 (lane S) or with a 200-fold excess of unlabeled nonspecific probe probe-neg (lane N). For EMSA of $AdpA_{lin}$ with m*adpAp*-B-2, concentrations of $AdpA_{lin}$ are 0, 1.6, and 3.2 μM, respectively.

Supplementary Table S1 Primers used in this study

**Primers**	**Sequence (5'-3')**
**Construction of *adpA$_{lin}$* mutants**	
ad-F1	GGCAAGCTTATCCTTGATCGGGTGGGACG
ad-R1	CTTCTAGACAGCCCATGTGGTGCGGTGAGT
ad-F2	CGTGGATCCGCGTTCGCCGAGTCCTGTC
ad-R2	CTGAATTCGATCATGCTTGGTCCCTCCCTG
ID*ad*-F1	GCGACGTGGACGATGATCAGGC
IDneo-R1	AGGGCTTCCCAACCTTACCA
IDneo-F2	GGGAAGGGACTGGCTGCTAT
ID*ad*-R2	GAACGGGAACAGCGGCAC
ad-C-F	CTTAGCCATATGAGCCACGACTCCACTGCC
ad-C-R	GGAGAATTCTGAGGATGGGACGGGTAAGGG
152-F	GCGTAAGGAGAAAATACCGCATCAG
152-R	TTCTGTGGATAACCGTATTACCGCC
**Catechol dioxygenase activity assays**	
xyl-F	ATGAACAGTGCCGGCTACGAGG
xyl-R	GGCCGATTCATTAATGCAGTCAGGTCAGCACGGTCATGAATC
adp-xyl-F	CTCTTCGCTATTACGCCAGGGTTCCCGTGGTGCTCCTCT
adp-xyl-R	CCTCGTAGCCGGCACTGTTCATACTGCTAAGCCCCCC
blp-xyl-F	CTCTTCGCTATTACGCCAGGGATCACCGTGTCCAGCCACTG
blp-xyl-R	CCTCGTAGCCGGCACTGTTCATCGAAGGGGCAGCGAGGTTTAAG
Up-xyl-F	CTCTTCGCTATTACGCCAGTGGCCTCATCGGGATAAGTGAGGCGG
Up-xyl-F	CCTCGTAGCCGGCACTGTTCATAAATTCGACCTCACCAC
Ap-xyl-F	CTCTTCGCTATTACGCCAGCCGTCTACGTCAGCATCGGG
Ap-xyl-R	CCTCGTAGCCGGCACTGTTCATGCGTCCACCACCATAAC
Cp-xyl-F	CTCTTCGCTATTACGCCAGGAAGGACGTCGAAGAGGTCACAGCG
Cp-xyl-R	CCTCGTAGCCGGCACTGTTCATGCCTCCGCCATCGGGTACCGGCC<br>CG
Dp-xyl-F	CTCTTCGCTATTACGCCAGCGACCATCCACTCCAGGAAGGCGA
Dp-xyl-R	CCTCGTAGCCGGCACTGTTCATTGTCCGCCGCTGTGACCTC
Jp-xyl-F	CTCTTCGCTATTACGCCAGCAGCCGTCGGCGTCGTCGTGGAGG
Jp-xyl-R	CCTCGTAGCCGGCACTGTTCATCATTGAATTCTCTTTCCTCACCAG
Kp-xyl-F	CCTCTTCGCTATTACGCCAGCGGTGGTGTCGAAGTGGAC
Kp-xyl-R	CCTCGTAGCCGGCACTGTTCATGCCACCGTCTCCCTGC
Vp-xyl-F	CCTCTTCGCTATTACGCCAGGTGTCTTGGAGTTCGATGATTTCCG
Vp-xyl-R	CCTCGTAGCCGGCACTGTTCATCTCGATGGCGTAAATGGCTCTCT<br>CC
Wp-xyl-F	CCTCTTCGCTATTACGCCAGGGACGTTCCACTCCGCACAGCGTGT

Wp-xyl-R	CCTCGTAGCCGGCACTGTTCATCATGGCACCTCGGTCCGTCTGAA GGGCT
**EMSA**	
Biotin-linker*	AGCCAGTGACGATAAG
n*ad*-F	AGCCAGTGACGATAAGGACGCTGACGGGTCCTTG
n*ad*-R	AGCCAGTGACGATAAGTCGGACGAGGACGACGACAACC
adp-A-F	AGCCAGTGACGATAAGCGCACCCTACGTGGATTGG
adp-A-R	AGCCAGTGACGATAAGTCTACTGTGTCGCGTGATGCC
adp-B-F	AGCCAGTGACGATAAGGGTTCCCGTGGTGCTCCTCTG
adp-B-R	AGCCAGTGACGATAAGGTGGTCGGAAGTTGGCTGAAAAG
adp-B-1-F	AGCCAGTGACGATAAGCGGGGACACACAGGGGTGGGC
adp-B-1-R	AGCCAGTGACGATAAGTGGGGACCCTTTCCGTGCAG
adp-B-2-R	AGCCAGTGACGATAAGCGCCCACCCCTGTGTGTCC
m*adp*-B-2-R	GGCCCGGGATATCCGATGGGGGGCCCGTTCTTCTATAGGTAGCCG TGCCGCACCGCG
blp-A-F	AGCCAGTGACGATAAGGCTCCCTCAGAACTCAATAAAATAAG
blp-A-R	AGCCAGTGACGATAAGCGTGTCTGCATTCCACCATC
blp-B-F	AGCCAGTGACGATAAGCGGCAAGTCCACGCTGAT
blp-B-R	AGCCAGTGACGATAAGGGTTGAACGCCTGGAAGAT
blp-N-F	AGCCAGTGACGATAAGCGACCACCTGGGTCTCGCC
blp-N-R	AGCCAGTGACGATAAGGGAACAGCGGCTGACTACACGG
m*blp*-B-F	AGCCAGTGACGATAAGCGGCAAGTCCACGCTGATGCACTGCGCT ATAGGCCTGGACAC
Up-A-F	AGCCAGTGACGATAAGAAGCAGCGATTCACCCC
Up-A-R	AGCCAGTGACGATAAGTGATCCGTACTTGATACATTGG
Up-B-F	AGCCAGTGACGATAAGCAATGTATCAAGTACGGAT
Up-B-R	AGCCAGTGACGATAAGGATTTGTTCTAGGTAAACG
m*Up*-B-R	CAGCTGTTGTTGCCGCTTTGGATGGTC
m*Up*-B-F	GCGGCAACAACAGCTGGGCTCATTTCATTC
Up-C-F	AGCCAGTGACGATAAGGCGTTTACCTAGAACAAATCCG
Up-C-R	AGCCAGTGACGATAAGGACGGAACCCTGGAGGAGAT
Ap-A-F	AGCCAGTGACGATAAGTCCACGCACCCAGACGCTTG
Ap-A-R	AGCCAGTGACGATAAGCGAGACAAGCAATCGGCTTCACG
m*Ap*-R	CGGGAGTCGGCGCGAACGGCCCTCGATATCCGGTGCAT
m*Ap*-F1	CGTTCGCGCCGACTCCCGGATAAC
Cp-A-F	AGCCAGTGACGATAAGGAAGGACGTCGAAGAGGTCACAGCG
Cp-A-R	AGCCAGTGACGATAAGCGTGCGGTCCAGCATGGGC
Cp-B-F	AGCCAGTGACGATAAGGCCCATGCTGGACCGCACG
Cp-B-R	AGCCAGTGACGATAAGGCGGTCCTCCAGGAACAGCAGACT
Cp-C-F	AGCCAGTGACGATAAGAGTCTGCTGTTCCTGGAGGACCGC

Cp-C-R	AGCCAGTGACGATAAGCCACATGGTGCGGGTCGG
Dp-A-F	AGCCAGTGACGATAAGCGTGGCAGGCGAACACATCG
Dp-A-R	AGCCAGTGACGATAAGCCTGCCATCGGACGGGGAC
Jp-A-F	AGCCAGTGACGATAAGGGCCACACGTCGTAGACATCTGCG
Jp-A-R	AGCCAGTGACGATAAGCGCCGTCCCGGTCGAAGAAC
mJp-A-R	GCTCGCCGAGCCTGGTGAGGAAAGAGAATTC
mJp-A-F	CCTCACCAGGCTCGGCGAGCGGCCGCAG
Jp-B-F	AGCCAGTGACGATAAGGCGACGGCAGGCCCTACCC
Jp-B-R	AGCCAGTGACGATAAGGGGGCGATGCCGAGCAGTTC
Kp-B-F	AGCCAGTGACGATAAGGGCGTAGACGGACACGTGGTAGCG
Kp-B-R	AGCCAGTGACGATAAGCACCCCACGGAGCTGGACCTG
m*Kp*-B-F	AGCCAGTGACGATAAGGCCCGGAACGCGGCGGCGGCG
m*Kp*-B-R	AGCCAGTGACGATAAGCTATAGGCTTCGCGGGCTTCGAACTCCGC AGGATATCCGCCGACTTCGACGGT
Vp-A-F	AGCCAGTGACGATAAGCGCAGGTGTCTTCCCTCGT
Vp-A-R	AGCCAGTGACGATAAGTACCTTTCCCTTGCGGTTCGC
**qRT-PCR**	
q*hrdB*-F	GGCAACCTCGGTCTGATCC
q*hrdB*-R	CGGGATACGGATGGTGC
q*bl*-F	GCCCGGAGCCGGACTTGAAC
q*bl*-R	GGATGGTGGAATGCAGACA
q*U*-F	CGCAACTACGCCTGGGTGG
q*U*-R	GGTGTCGGACTTCTTCTGCCC
q*A*-F	CGACACCGCAAGCCTTCTCCGAT
q*A*-R	CGAGCAACCGCAGCCAGCCAC
q*C*-F	CCCCGCTGCACTTCGACGTGTC
q*C*-R	CGGATAGGGCACGGAGAGCCATAC
q*D*-F	CGAACGTCAGATGCGTGCGGT
q*D*-R	TGGCGAAGAGGGTCGGTGTC
q*J*-F	AGCGACGGGATCGTGTTTG
q*J*-R	CCTGGTCCTTCAGTGCCTCA
q*K*-F	TTCTTCACCTGCCTCCACG
q*K*-R	GGTAGCCCCGGTCGATCC
q*V*-F	CCCACCAGCACCGTCATG
q*V*-R	TGACCCGTGAGCAGTTCGTG
q*W*-F	CGGTTCCCGCACCAGAAGA
q*W*-R	GCTGCGTGAGGACGTGGATG
